# Effect of *b* Value on Imaging Quality for Diffusion Tensor Imaging of the Spinal Cord at Ultrahigh Field Strength

**DOI:** 10.1155/2021/4836804

**Published:** 2021-01-06

**Authors:** Shu-Sheng Bao, Can Zhao, Xing-Xing Bao, Jia-Sheng Rao

**Affiliations:** ^1^Beijing Key Laboratory for Biomaterials and Neural Regeneration, Department of Biomedical Engineering, School of Biological Science and Medical Engineering, Beihang University, Beijing 100083, China; ^2^Institute of Rehabilitation Engineering, China Rehabilitation Science Institute, Beijing 100068, China; ^3^Beijing Advanced Innovation Center for Biomedical Engineering, Beihang University, Beijing 100083, China

## Abstract

**Objective:**

To explore the optimal *b* value setting for diffusion tensor imaging of rats' spinal cord at ultrahigh field strength (7 T).

**Methods:**

Spinal cord diffusion tensor imaging data were collected from 14 rats (5 healthy, 9 spinal cord injured) with a series of *b* values (200, 300, 400, 500, 600, 700, 800, 900, and 1000 s/mm^2^) under the condition that other scanning parameters were consistent. The image quality (including image signal-to-noise ratio and image distortion degree) and data quality (i.e., the stability and consistency of the DTI-derived parameters, referred to as data stability and data consistency) were quantitatively evaluated. The min-max normalization method was used to process the calculation results of the four indicators. Finally, the image and data quality under each *b* value were synthesized to determine the optimal *b* value.

**Results:**

*b* = 200 s/mm^2^ and *b* = 900 s/mm^2^ ranked in the top two of the comprehensive evaluation, with the best image quality at *b* = 200 s/mm^2^ and the best data quality at *b* = 900 s/mm^2^.

**Conclusion:**

Considering the shortcomings of the ability of low *b* values to reflect the microstructure, *b* = 900 s/mm^2^ can be used as the optimal *b* value for 7 T spinal cord diffusion tensor scanning.

## 1. Introduction

Magnetic resonance imaging (MRI) is an important imaging method to detect spinal cord injury (SCI) at present, but conventional MRI has deficiencies in detecting the microstructure of spinal cord tissue and the integrity of white matter fiber tracts [1]. Diffusion tensor imaging (DTI), a special MRI technique, can reflect the alterations of tissue microstructure by measuring the water diffusion motion, so it has important application prospects in evaluating the severity of SCI and its therapeutic effects [1–3]. At present, there are many researches on SCI using DTI technology. Zhao et al. [4] used spinal cord contused Wistar rats to analyze the DTI data and behavioral scores before SCI, and 1, 3, 7, 14, and 84 days post SCI. It is proved that DTI technique can noninvasively reflect the spatiotemporal characteristics of SCI-induced white matter fiber bundles degeneration and can directly show the damage of white matter fiber tracts. Liu et al. [5] investigated the dynamic correlation of DTI and neurological function scores in spinal cord-injured beagles and showed that DTI has the potential to accurately predict the recovery of neurological function after SCI.

During scanning, the setting of sequence parameters will affect the DTI results, and the diffusion sensitivity coefficient *b* value is one of them. Chung et al. [6] assessed the parameter setting of DTI in normal human brain and revealed that the increase of *b* value generally leads to the decrease of signal-noise ratio (SNR) of DTI image. Barrio-Arranz et al. [7] performed brain DTI on 13 healthy subjects and found that the increase of *b* value would lead to the decrease of mean diffusivity (MD), axial diffusivity (AD), and radial diffusivity (RD) when the gradient directions and voxel resolution remained constant. Therefore, to some extent, the setting of *b* value will determine the reliability of DTI results.

At present, many studies focus on the exploration of the optimal *b* value in DTI sequence at the low field strength of MR scanner. Sakai et al. [8] evaluated the DTI imaging quality of lumbar nerve root at *b* = 200, 400, and 800 s/mm^2^ by using 1.5 T MRI and reported the less distortion of DTI images at 400 s/mm^2^. Taib et al. [9] performed a human whole brain DTI at 1.5 T scanner and used two settings of voxel size: 2.0 × 2.0 × 2.0 mm^3^ and 2.5 × 2.5 × 2.5 mm^3^. Six sets of parameter settings were formed under three *b* values: 700, 1000, and 1200 s/mm^2^. In the comparison of SNR, fractional anisotropy (FA), and MD under these six settings, it is suggested that the SNR, FA, and MD of imaging results are the best when the voxel size is 2.5 × 2.5 × 2.5 mm^3^, and the *b* value is 700 or 1000 s/mm^2^. Although there have been some achievements in the study of optimal *b* value settings at low field strength, researchers often need to use the ultrahigh field strength MR equipment in the basic research of SCI to obtain precise DTI images [10, 11]. However, it is still unclear whether the optimal *b* value obtained at the low field strength (1.5 T) can accurately guide that at the ultrahigh field strength. Therefore, it is essential to explore the most appropriate *b* value in the DTI sequence under the ultrahigh field strength.

In this study, the DTI data of normal and spinal cord-injured animals were collected by using the ultrahigh field strength (7 T) MR instrument. Image quality (including image SNR and image distortion degree) and data quality (including data stability and data consistency) were calculated under different *b* value settings. The optimal *b* value setting for spinal cord DTI under the 7 T scanner was explored and evaluated through these four indicators.

## 2. Materials and Methods

### 2.1. Data Acquisition

#### 2.1.1. Animals

Fourteen adult female Wistar rats (weighed between 180 and 220 g, 5 normal and 9 spinal cord transected) suffered an MRI scan. All experiment procedures were approved by the Biological and Medical Ethics Committee of Beihang University.

#### 2.1.2. Image Acquisition

All datasets were collected in vivo by using the Bruker BioClinScan Animal MRI System (bore size 31 cm, gradient field strength 290 mT/m, and slew rate 1160 T/m/s) dedicated to small animals. Rats were anesthetized by intraperitoneal injection of ketamine (60 mg/kg) and xylazine (10 mg/kg). Anesthetized animals were placed in the supine position, fixed with foam pads and adhesive tapes located in the forelimbs, hindlimbs, and abdomen. A dual-channel surface receive coil was fixed on the back corresponding to the thoracic spinal cord, and the head was lifted by a head sheath to reduce the amplitude of respiratory movement and to avoid obvious movements during scanning. The midline of the spinal cord was approximately parallel to the axis of the magnet. Three-plane loc scanning was performed to obtain the position of the region of interest (ROI). Saturation bands were added to the thorax and abdomen to reduce motion artifacts during scan. Parallel imaging was not used during scan.

T2-weighted structural images were acquired using rapid acquisition with relaxation enhancement (RARE) sequence. The parameters were as follows: TR/TE = 3000/45 ms, FOV = 4 × 4 cm^2^, matrix = 256 × 256, slice thickness = 1 mm without interslice space, number of slices = 20, NEX = 6, and phase encode direction is left-right (TR: repetition time; TE: echo time; FOV: field of view; NEX: number of excitations).

Axial-orientation diffusion-weighted (DW) images were acquired at the same central line as T2-weighted images. Single-shot spin-echo echo-planar imaging (SE-EPI) sequence was used with the following parameters: TR/TE = 5000/23 ms, FOV = 1.88 × 1.88 cm^2^, matrix = 128 × 128, number of slices = 20, interslice space = 0, voxel size = 0.147 × 0.147 × 1 mm^3^, NEX = 4, and phase encode direction is left-right. Considering the anesthesia effect and the requirement of spinal cord DTI, to shorten the scanning time, gradient directions were set to six noncollinear directions. In the same scanning parameters mentioned above, b0 was set to 0 s/mm^2^, and the *b* values were set to 200, 300, 400, 500, 600, 700, 800, 900, and 1000 s/mm^2^, respectively. The effective *b* values were about: 206, 309, 410, 513, 613, 715, 816, 919, and 1020 s/mm^2^, respectively. All data under each *b* value were collected for each animal, and the scanning order of *b* values was randomly selected. The scanning time for each *b* value was ~3 minutes, and the whole scanning time for each animal was ~40 minutes. The respiratory rate (~70 breaths/min) and heart rate (~270 beats/min) of the animals remained stable throughout the scanning process.

All collected images were first screened by visual inspection to exclude data with obvious artifacts that may affect the accuracy of subsequent image processing results.

#### 2.1.3. DTI Processing

The professional software MedINRIA (http://www-sop.inria.fr/asclepios/software/MedINRIA) was used to process and calculate the DTI data under each *b* value by the methods below: first, the DICOM volumes in mosaic format were split, and the series were averaged according to their gradient directions. Then, eddy current correction was performed for all DW scans using 12-mode linear affine intrasubject registration with the b0 image as a reference. The method proposed by Ardekani and Sinha [12] was used to correct the geometric distortions caused by residual susceptibility. Anisotropic filter was used for image smoothing and denoising. For each session, intensity histograms between nonweighted EPI images and T2-weighted structural images were normalized and matched, and nonrigid deformation fields were estimated to register EPI to the structural volume. For each direction in all DW scans, the deformation field was calculated in the same way and applied accordingly. After the postprocessing, ROIs were selected from the middle slice of the spinal cord and 5 mm and 10 mm rostral and caudal from the middle slices (i.e., the 1st, 5th, 10th, 15th, and 20th slices of the images). FA and MD values were then extracted from the ROIs for each animal [13].

### 2.2. Evaluation Indicators

#### 2.2.1. Image Quality Indicators

EPI images in the fourth gradient direction were randomly selected from six noncollinear gradient directions for image quality evaluation. The SNR and the degree of distortion of these images were calculated, respectively. The slices of images are the same as where FA and MD were measured.

**Figure 1:**
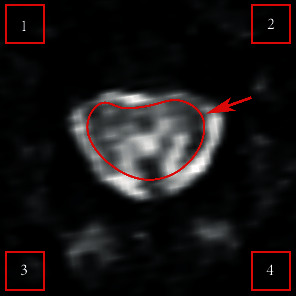
Schematic diagram of ROI selection in the axial EPI images of the spinal cord. Arrow indicated the central signal area (spinal cord), and the four square ROIs are boundary signal areas (background noise).

*(1) SNR:* ROI was selected manually at the central part of the spinal cord, and four square ROIs were taken at the background area, whose edges were 0.147 mm away from the boundary, and the area was 2.161 mm^2^ for each square ROI (Figure 1). The averaged signal intensity of the ROI at the center ($\bar{S}$) and the mean standard deviation in the signal intensity of the square ROI at the edge ($\overset{¯}{SD}$) were extracted and calculated by formula [14]

(1) $$\begin{array}{c} \text{SNR}=0.655\times \frac{\overset{¯}{S}}{\overset{¯}{SD}}. \end{array}$$

The factor 0.655 is due to the Rician distribution of the background noise in an MRI image [14]. The larger the SNR, the more obvious the contrast between the useful signal and the interference noise, that is, the better the image qualities.

*(2) Degree of Image Distortion:* T2-weighted structural images and corresponding EPI images were selected. Normalized mutual information (NMI) method was used to evaluate the distortion degree of EPI images [15]. Image mutual information values were calculated as follows:

(2) $$\begin{array}{c} I\left(A,B\right)=H\left(A\right)+H\left(B\right)-H\left(A,B\right). \end{array}$$

And normalization processing was performed using the formula as follows:

(3) $$\begin{array}{c} \text{NMI}=2\text{I}\left(\text{A},\text{B}\right)/\left(\text{H}\left(\text{A}\right)+\text{H}\left(\text{B}\right)\right), \end{array}$$

where *H* (*A*) represents the information entropy of *A*, and *H* (*A*, *B*) is the combined entropy of *A* and *B* [16]. In this study, the larger the NMI value, the smaller the distortion degree of the EPI images relative to the corresponding T2-weighted structural images, that is, the better the quality of the EPI images.

#### 2.2.2. Data Quality Indicators

**Figure 2:**
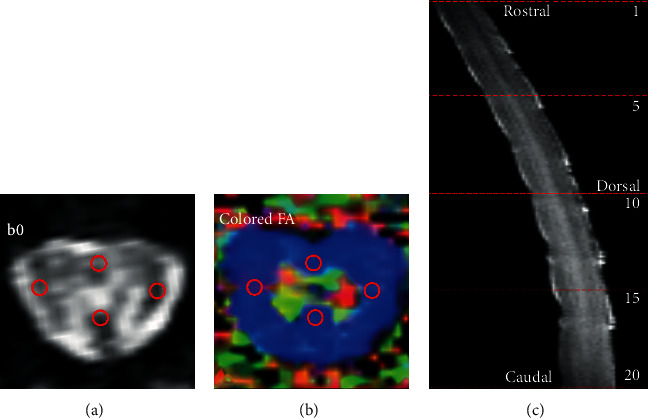
ROI diagram of FA and MD values. (a) b0 image and the four selected ROIs (red circles); (b) display of ROI position on the corresponding colored FA image; (c) sagittal T2-weighted structural image of the spinal cord, with dashed lines indicating the location of the five slices of the spinal cord where the above measurements were executed.

Based on the b0 images (*b* value = 0 s/mm^2^), four suitable ROIs were selected at both ends of the sagittal and transverse diameters of the spinal cord with an area of 0.086 mm^2^ (Figure 2). FA and MD values in the ROI were extracted and calculated.

*(1) Data Stability:* compare the overall data stability of FA and MD values, respectively, to measure imaging quality. The magnitude of individual data deviating from the mean value of this slice's overall data is calculated. The smaller the deviation, the smaller the difference of data in the same slice, that is, the better the data stability.

Take FA as an example: calculate the absolute value (|∆*FA*_*i*_|) of the difference between each slice's FA value (*FA*_*i*_) and mean FA value ($\bar{FA}$) for each rat under each *b* value, and take the mean of all absolute values as the FA stability (*FA*_*s*_) under this *b* value, i.e.,

(4) $$\begin{array}{c} {FA}_{s}=\bar{\left|{FA}_{i}-\bar{FA}\right|}. \end{array}$$

Same for the MD stability (*MD*_*s*_).

In principle, stability is a measure of the difference of data in the same slice, which will not be affected by the nature of data itself, and the changing trend of FA and MD stability with *b* value may be different. Therefore, we chose to integrate FA and MD stability as the data stability indicator by the methods below: normalize the FA and MD values to the [0, 1] interval with the min-max normalization method. By summing the normalized FA and MD values (*nFA*_*s*_ and *nMD*_*s*_) and normalizing the result again, we get the data stability (Data_*s*_) of this slice, i.e.,

(5) $$\begin{array}{c} {\text{Data}}_{s}=\text{minmax}\left({nFA}_{\text{s}}+{nMD}_{s}\right). \end{array}$$

*(2) Data Consistency:* compare the variation of the data in the 1st, 5th, 10th, 15th, and 20th slices to evaluate the consistency of FA and MD values, respectively, between the different slices. Variability in mean FA and MD values between different slices were assessed, respectively. The smaller the variability, the smaller the variation of data between different slices, that is, the better the consistency. Since the data of different slices differ greatly in SCI rats, this indicator is only for healthy animals [4, 11].

Take FA as an example: calculate the absolute value ($\left|∆\bar{FA}\right|$) of the difference in each slice's mean FA value ($\bar{FA}$) for each rat under each *b* value, and take the mean of all absolute values as the FA consistency (*FA*_*c*_) under this *b* value. Same for the MD consistency (*MD*_*c*_).

See for data stability: consistency is a measure of the variation of data between different slices, which will not be affected by the nature of data itself, and the changing trend of FA and MD consistency with *b* value may be different. Therefore, we chose to integrate FA and MD consistency as the data consistency indicator by the formula

(6) $$\begin{array}{c} {\text{Data}}_{c}=\text{minmax}\left({nFA}_{c}+{nMD}_{c}\right). \end{array}$$

#### 2.2.3. Statistical Analysis

The results for each indicator were statistically analyzed using spss22.0 (SPSS Inc., Chicago, IL). Firstly, the normality of the results was tested. Then, the paired sample *T* test (normal distribution) or Wilcoxon signed rank test (nonnormal distribution) were used to compare the differences of each indicator under different *b* values, and Bonferroni correction was used for multiple comparisons. Mann-Whitney *U* test was used to compare the difference between healthy and SCI groups at the same *b* value. Pearson's correlation analyses were conducted to explore the correlations between image/data quality results and *b* values. The level of statistical significance was set at *P* < 0.05 (for Bonferroni multiple comparison correction: *P*_corrected_ < 0.05/*n*, where *n* is the number of tests), and the results are given as mean ± standard deviation (mean ± SD).

### 2.3. Comprehensive Evaluation

In order to comprehensively evaluate the influence of different *b* values on the imaging quality, the indicators of image quality (including SNR and distortion degree) and data quality (including data stability and consistency) were processed as follows: (i) SNR, NMI, stability, and consistency results of each rat under each *b* value were normalized by using min-max normalization method. (ii) The normalized values were integrated. SNR and NMI values were added as a result of image quality; data stability and consistency values were also added as a result of data quality (DQ) (i.e., DQ = Data_*s*_ + Data_*c*_). (iii) The results of image quality and data quality of each *b* value were normalized again and then used as the horizontal and vertical coordinates of the two-dimensional plane. (iv) The comprehensive imaging quality is evaluated by comparing the Euclidean metric (EM) between the different *b* value coordinates and the axis origin. The larger the EM is, the better the integrated imaging quality of the *b* value is.

## 3. Results

DTI datasets of 5 healthy and 9 spinal cord-injured rats under different *b* values were collected, and the corresponding T2-weighted structural images of each rat were also obtained. No structural image or DTI dataset was excluded.

### 3.1. Image Quality Evaluation Results

**Figure 3:**
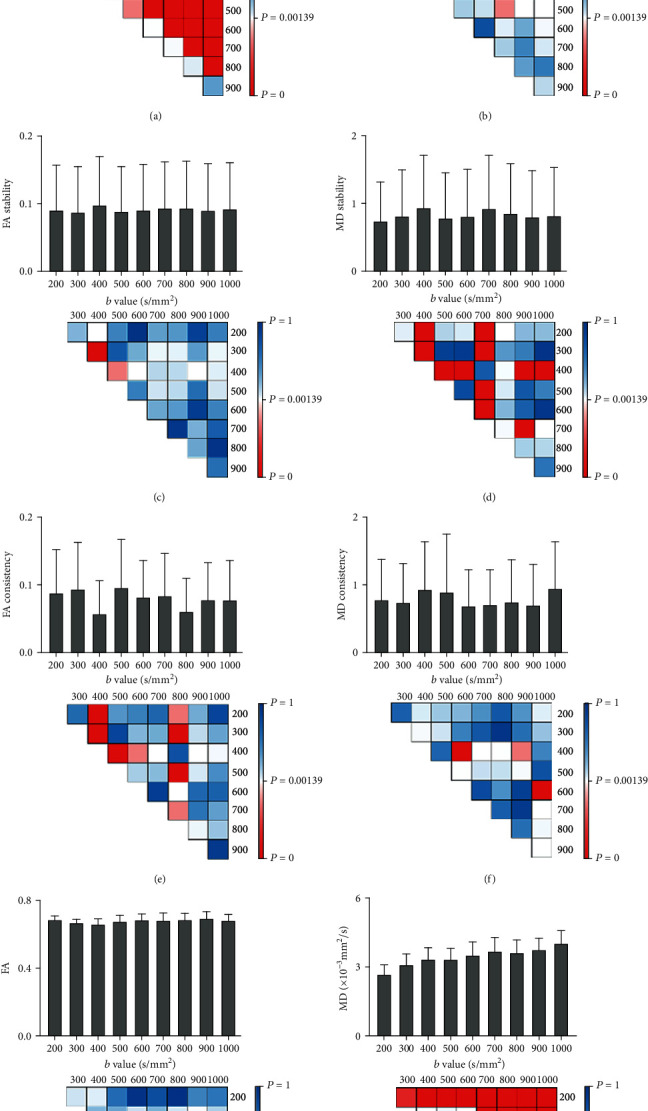
Color matrix diagram of the results and statistical differences of each indicator. (a–h): SNR, NMI, FA stability, MD stability, FA consistency, MD consistency, FA, and MD, respectively. Data results are given as mean ± SD. Color bar indicates the *P* value, and red color represents a significant difference.

The SNR of the image gradually decreases with the increased *b* values. The SNR result decreased from 6.0 ± 1.6 (*b* = 200 s/mm^2^) to 3.6 ± 1.2 (*b* = 1000 s/mm^2^), and there were significant differences among all groups (*P*_corrected_ < 0.00139) except for the *b* value between 600 vs. 700 (*P*_corrected_ = 0.010), 700 vs. 800 (*P*_corrected_ = 0.017), 800 vs. 900 (*P*_corrected_ = 0.052), and 900 vs. 1000 (*P*_corrected_ = 0.328) (Figure 3(a)).

The highest NMI result of the image appeared at *b* = 200 s/mm^2^ (0.381 ± 0.038) and the lowest at *b* = 1000 s/mm^2^ (0.354 ± 0.044). Comparison results showed that there were significant differences among group *b* = 200, 300 s/mm^2^ and group *b* = 600, 700, 800, 900, 1000 s/mm^2^, group *b* = 400 s/mm^2^ and group *b* = 800, 900, 1000 s/mm^2^, group *b* = 500 s/mm^2^ and group *b* = 800 s/mm^2^ (for all, *P*_corrected_ < 0.00139) (Figure 3(b)).

### 3.2. Data Quality Evaluation Results

For data stability, the difference in FA stability between groups was small. Only FA stability values at groups *b* = 300 s/mm^2^ (0.088 ± 0.067) and *b* = 500 s/mm^2^ (0.089 ± 0.067) were significantly better than that at group *b* = 400 s/mm^2^ (0.099 ± 0.071) (300 vs. 400: *P*_corrected_ = 0.000; 400 vs. 500: *P*_corrected_ = 0.001) (Figure 3(c)). MD stability values at groups *b* = 400 s/mm^2^ (1.0 ± 0.8) and *b* = 700 s/mm^2^ (1.0 ± 0.8) were significantly worse than those at other groups (for all, *P*_corrected_ < 0.00139) (Figure 3(d)).

For data consistency, the FA consistency value at groups *b* = 400 s/mm^2^ (0.058 ± 0.048) and *b* = 800 s/mm^2^ (0.061 ± 0.049) was significantly better than that at other groups (for all *P*_corrected_ < 0.00139) (Figure 3(e)). However, the consistency of MD at group *b* = 400 s/mm^2^ (1.0 ± 0.7) was indeed worse than that at groups *b* = 600 s/mm^2^ (0.7 ± 0.5) and *b* = 900 s/mm^2^ (0.7 ± 0.6) (400 vs. 600: *P*_corrected_ = 0.000; 400 vs. 900: *P*_corrected_ = 0.001). The MD consistency at group *b* = 1000 s/mm^2^ (1.0 ± 0.7) was also pronounced worse than that at group *b* = 600 s/mm^2^ (*P*_corrected_ = 0.000). There was no significant difference among the other groups (for all, *P*_corrected_ > 0.00139) (Figure 3(f)).

### 3.3. Comprehensive Evaluation

**Figure 4:**
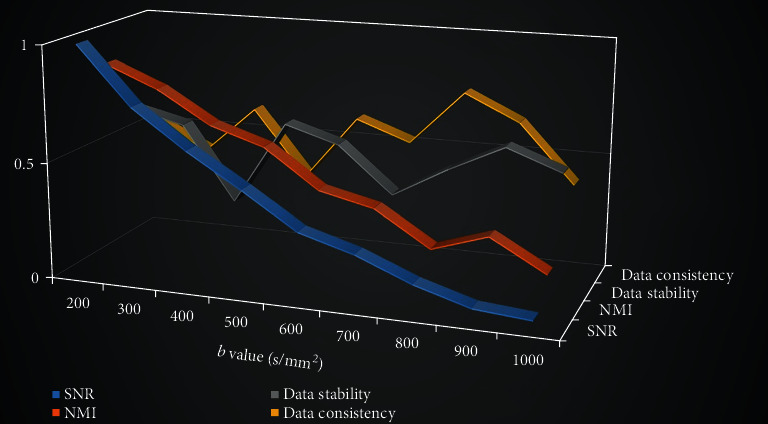
Three-dimensional line chart of the changes of four indicators under different *b* values. Different colors represented different indicators.

**Figure 5:**
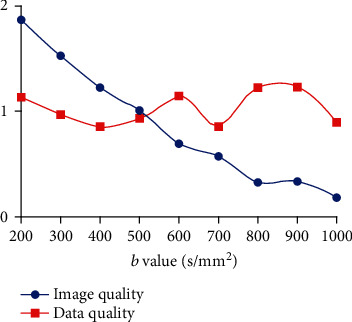
Tendency of image quality results composed of SNR and NMI, as well as data quality results composed of data stability and consistency under different *b* values.

As the *b* value increased, the SNR and NMI results decreased gradually (Figure 4). The image quality, as a combination of SNR and NMI, also showed a decline tendency (Figure 5) and was negatively correlated with *b* values (*r* = −0.9779, *P* < 0.0001, two-tailed). However, the data stability and consistency results showed relatively large fluctuations among the *b* values (Figure 4), and the overall data quality results reached the highest level at *b* = 800 and 900 s/mm^2^ (Figure 5). No significant correlation between data quality and *b* values was observed (*r* = 0.1469, *P* = 0.7061, two-tailed).

**Figure 6:**
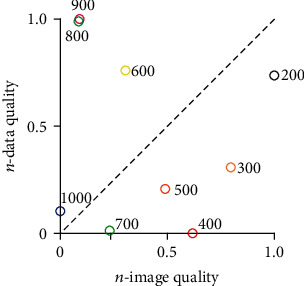
Distribution of imaging quality under each *b* value in two-dimensional coordinates. The 45° dashed line indicates that the *n*-image quality is the same as the *n*-data quality.

Figure 6 displayed the distribution of each *b* value in a two-dimensional plane composed by normalized image quality and data quality (*n*-image/*n*-data quality). The coordinates of low *b* value are distributed below the 45° dashed line, indicating they have better *n*-image quality than *n*-data quality. On the contrary, the high *b* value coordinates are distributed above the dashed line. The EM between the point at *b* = 200 s/mm^2^ and the axis origin was the longest (EM = 1.242584), followed by *b* = 900 s/mm^2^ (EM = 1.004105) and *b* = 800 s/mm^2^ (EM = 0.994274). Considering the insufficient sensitivity of low *b* value for the detection of microstructure [17, 18], *b* = 900 s/mm^2^ can be thought of as the most appropriate *b* value for rat spinal cord DTI scan at 7 T.

### 3.4. FA and MD Comparison

#### 3.4.1. Comparison between Different *b* Values

For FA, there was no significant difference in FA value for each *b* value, only group *b* = 400 s/mm^2^ was significantly lower than group *b* = 900 s/mm^2^ (*P*_corrected_ = 0.00132) (Figure 3(g)). For MD, when *b* value is not so high (*b* = 200 − 600 s/mm^2^), MD value increases with the increase of *b* value significantly (e.g., group *b* = 200 s/mm^2^ is significantly lower than group *b* = 300 − 1000 s/mm^2^, and group *b* = 300 s/mm^2^ is significantly lower than group *b* = 700 − 1000 s/mm^2^, 5), while with the gradual increase of *b* value (*b* = 700 − 1000 s/mm^2^), the increase of MD value is not significant anymore (except for the group *b* = 800 s/mm^2^, which was significantly lower than group *b* = 1000 s/mm^2^, *P*_corrected_ = 0.00028) (Figure 3(h)).

#### 3.4.2. Comparison between Healthy and SCI Groups

**Figure 7:**
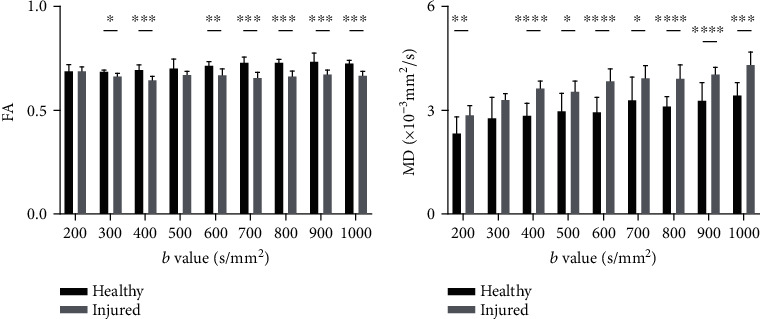
The differences in FA and MD obtained at different *b* values and in healthy and spinal-cord injured groups. ^∗^*P* < 0.05, ^∗∗^*P* < 0.01, ^∗∗∗^*P* < 0.005, ^∗∗∗∗^*P* < 0.001.

For FA value, there were significant differences between healthy and SCI groups at each *b* value, except at *b* = 200 s/mm^2^ (*P* = 0.699) and *b* = 500 s/mm^2^ (*P* = 0.190). Similarly, for MD value, we found significant differences between the two groups except when *b* = 300 s/mm^2^ (*P* = 0.060) (Figure 7).

## 4. Discussion

In this study, rats' spinal cord DTI was performed under ultrahigh field strength (7 T) MR equipment. A series of *b* values were set, and the imaging quality under each *b* value was analyzed from two aspects: image quality and data quality. Although different hardware and research purposes may affect the user's choice, there are rules for the effect of different *b* values on imaging quality. By comparing the results of a series of *b* values in the spinal cord DTI under the same scanning conditions, the influence of *b* values on image quality and data quality was revealed. The optimal *b* value in rats' spinal cord scanning was determined by integrating four indicators, thus providing guidance for the parameter settings of animal experiments with ultrahigh field intensity MR equipment.

Data reliability is one of the priority aspects to be considered in diffusion imaging quantitative studies. In order to improve the data reliability as much as possible, a variety of quality control methods were used in this study. In the step of setting scanning parameters, we used a water phantom to conduct sufficient preexperiments, ensuring the correct application of diffusion weighting gradients for each *b* value and determining the appropriate setting for each parameter [19, 20], such as ss-SE-EPI sequence with a fast acquisition speed, which can acquire an image within a very short time and thus reduce the influence of physiological motion (e.g., breathing) [21]. In the data processing, we also used a variety of postprocessing methods, like eddy current correction, anisotropic smoothing, and motion correction [22–24]. Through these quality control procedures, the impact of equipment, animal physiological structure, and physiological activities on data reliability was reduced as far as possible.

As one of the basic parameters of image quality, SNR is an important criterion to evaluate medical image quality [25, 26]. Previous studies have shown that high *b* value images usually tend to have a decrease in SNR due to the application of large diffusion gradients [6]. In this research, SNR of the images at *b* = 300 − 1000 s/mm^2^ is significantly lower than that at *b* = 200 s/mm^2^, which is consistent with the previous studies [27, 28]. We also noticed that the SNR at high *b* values was relatively low in this study, which means relatively high noise in the image, making it insufficient to quantify right FA and MD according to Jones and Basser [29]. However, the reason why low SNR leads to the wrong estimation of FA and MD is mainly due to the influence of noise floor sampling [29]. But this had little effect on this experiment, as the data quality was evaluated by the FA/MD stability and consistency rather than the FA/MD value. Even if a low SNR magnified the impact of noise floor sampling on FA/MD at a certain *b* value, the influence would be same for FA/MD at all locations in the image at that *b* value, thus has little impact on the FA/MD stability and consistency. Furthermore, Landman et al. [30] suggested that the propensity for bias and errors did not monotonically increase with noise. Giannelli et al. [31] compared the DTI measurements of two coils with different SNR and found that the difference in mean FA and MD values between the two coils was relatively significant, while the standard deviation of FA and MD only showed a trend of variation, indicating that the distribution of the measured DTI-derived parameters was relatively less affected by SNR. Finally, it is known that SNR will decrease with the increased *b* value, even if this will impact FA and MD values; it is also one of the consequences by increasing *b* values. Only from the perspective of SNR, the choice of the optimal *b* value should be as small as possible. However, low *b* value DTI results lack to reflect the microstructure. Fukutomi et al. [17] found that DTI-derived data at low *b* value cannot reasonably characterize the microstructure of the cerebral cortex. Thapa et al. [32] displayed that the gray-white matter contrast of spinal cord DTI is more obvious at high *b* values than that at low *b* values. For these reasons, a lower *b* value cannot be selected just to obtain a better image SNR.

Previous studies on optimal *b* value are mainly focused on the low field strength. Taib et al. [9] also evaluated the imaging quality from the aspects of image quality (SNR) and data quality (FA, MD). However, the number of *b* values set in their study was relatively less (*b* = 700, 1000, and 1200 s/mm^2^), and only SNR, FA, and MD were assessed. In our study, a series of *b* values were set, and the NMI indicator was also added. The use of NMI to assess image distortion mainly takes into account the following characteristics of the spinal cord relative to the brain [22]: (i) the spinal cord has a narrow physiological structure; (ii) adjacent vertebrae and intervertebral discs of the spinal cord may cause magnetic field inhomogeneity; and (iii) spinal cord is easily affected by physiological activities. These factors can lead to serious distortion of EPI images during DTI scanning. For these reasons, measuring the degree of distortion is essential to evaluate the imaging quality of spinal cord DTI. The differences in the morphological structure and physiological environment between the brain and the spinal cord may also be one of the reasons why the *b* values commonly used in the studies of brain DTI studies are different from those of spinal cord DTI.

At present, the evaluation of DTI data quality mostly focuses on the changing trend of indexes such as FA and MD. For example, some research results show that the MD value decreases with the increasing of *b* value [33–35], while the FA value is less affected by the alteration of *b* value [34, 35]. In our study, the difference in FA value under different *b* values was small, which was consistent with these results. However, the changing trend of MD value is not the same, which should be noted as the enhancement of noise floor effects [36] and the attenuation of interference of nondiffusion effects [37] usually lead to lower MD value at high *b* values. While in this study, it was found that MD value increased with the increasing of *b* value, then it tended to be stable. After confirming that it was not an erroneous result caused by operational lapses, we made the conjecture that the anomaly was attributed to the field strength and *b* value setting.

Generally, high field strength is beneficial for the detection of subtle diffusion in tissues and for the precise estimation of DTI-derived indices, because it can bring about a relatively high SNR [6], which may compensate to some extent for the decreased SNR caused by the increased *b* value. At the ultrahigh field strength (7 T) set up in this study, subtle diffusion which was difficult to detect at low field strengths (1.5 T, 3 T) may be captured and used for the estimation of DTI-derived indices with the increasing of *b* value (i.e., diffusion sensitivity coefficient), leading to the slight increase of MD.

Second, Bastin et al. [38] and Froeling et al. [39] showed that a low SNR generally lead to the overestimation of the primary eigenvalue *λ*_1_ and the underestimation of the other two eigenvalues *λ*_2_, *λ*_3_, which finally caused a lower estimated MD than its true value. However, considering the narrow physiological structure of spinal cord, in which the diffusion of water molecules is overwhelmingly along the direction of white matter fiber tracts [38], results in the contribution of *λ*_1_ to MD value in spinal cord DTI much greater than that of *λ*_2_ and *λ*_3_, which may have led to the phenomenon that MD did not suffered an obvious underestimation in this experiment despite the decreasing SNR with increasing *b* value.

Meanwhile, the range of *b* value setting in this research (200-1000 s/mm^2^) was much smaller than those other studies (such as the 500-2500 s/mm^2^ in Wu et al. [33] and 350-3000 s/mm^2^ in Dudink et al. [34]), with the highest *b* value of 1000 s/mm^2^, while nonmono-exponential diffusion decay at *b* > 1000 s/mm^2^ had been observed in rabbit [40] and rat [41] hearts, implying the possible nondiffusion effects. And Scott et al. [42] showed that the reference *b* value (i.e., diffusion weighting of the reference images) would also affect the changing trend of MD as a function of *b* value, as the application of proper reference *b* value could effectively suppress the influence of tissue capillary perfusion, thereby making MD less dependent on the *b* value. Therefore, the *b* value setting in this experiment may not completely suppress the nondiffusion effects, which may have led to the fact that MD did not decrease with the increasing of *b* value. The study of Wang et al. [43] can verify our conjecture to some extent; they performed the DTI of rats' knee joint under 9.4 T ultrahigh field strength and set a series of *b* values of 250, 500, 750, 1000, and 1250 s/mm^2^. Comparing MD results under different parameter settings, it is found that MD value increases with the increasing of *b* value, but when *b* value is higher than 750 s/mm^2^, MD value tends to be stable, suggesting that too high *b* value may not have much effect on the improvement of DTI data quality. Our results also showed that when *b* value was in low level (*b* = 200 − 600 s/mm^2^), MD value increased with the increasing of it; however, MD was in stable when *b* value was in high level (*b* = 700 − 1000 s/mm^2^). The data quality at *b* = 1000 s/mm^2^ is also not better than that at *b* = 900 s/mm^2^, which are consistent with the results of previous studies.

In the present study, the FA value of SCI group was generally lower than that of healthy group at each *b* value, while the MD value was on the contrary, mostly higher than that of healthy group, keeping consistent with some previous studies [44, 45]. This may be due to the SCI-induced disruption of the normal physiological structure of spinal cord, such as axonal interruption and disintegration which may reduce the difference in water molecules' diffusion along axonal direction and other directions, leading to the reduction of FA value. And the retention of cerebrospinal fluid (CSF) at the injury site may increase the diffusion degree of water molecules, resulting in the increase of MD value. There are also some studies on acute SCI that indicate a transient decrease in MD value postinjury [5, 46], which may be the result of cell swelling, as it can cause an increase in cell radius, leading to an increased tortuosity, and ultimately induce a decrease in MD value [5].

While the differences in FA and MD obtained at different *b* values and in different groups have been widely evaluated nowadays, there are relatively few studies on the stability and consistency of these data in the same individual. The results of this study showed the *b* value-induced obvious fluctuations of FA and MD values in the same slice and between different slices. It is suggested that further studies should also pay attention to the effects of such changes on the experimental datasets.

Our study showed that image quality is generally better than data quality with lower *b* values, while the opposite is true with higher *b* values. The main reason for this phenomenon is that, on the one hand, image quality is negatively correlated with *b* value, meaning the significant decrease of image quality with the increase of *b* value; on the other hand, data quality is independent of the change of *b* value. This result further illustrates that the trade-off between image quality and data quality is very important. It is of great significance to obtain more accurate DTI results by setting and optimizing imaging parameters to meet the needs of image quality and improve the data quality as much as possible.

There are some limits in this study. First, the evaluation criteria for image quality and data quality include, but are not limited to, the four indicators used here. Whether the current research results can be applied to other evaluation environments needs further verification. In addition, considering that too high *b* value will lead to a significant reduction of SNR and a lack improvement of data quality in DTI, subsequent studies can appropriately reduce the range of *b* value and refine the division of *b* value to obtain more accurate results. Thirdly, the number of gradient directions (NGD) selected in this experiment is relatively small, while more directions are generally used in the clinical examinations to improve DTI reliability [47]. However, the main purpose in this study is to explore the difference in DTI imaging quality under different *b* values and find the optimal *b* value setting; the conclusion will mainly be affected by *b* values rather than other parameters like NGD. Previous experiments have also shown that DTI can provide measurements with high reliability even for images with few directions [48], and six-direction data provide diffusion measures with comparable robustness to those under higher gradient directions [49].

## 5. Conclusion

This study explored the effect of different *b* values on the imaging quality of spinal cord DTI at 7 T Bruker MR instruments, and proved that *b* = 900 s/mm^2^ had the best comprehensive results of image quality and data quality. The results of this research provide an experimental basis for the future extensive application of DTI in the study of SCI and are helpful for researchers to set imaging scanning parameters more reasonably.

## Data Availability

The data used to support the findings of this study are available from the corresponding author upon request.
